# *E. coli* O157 on Scottish cattle farms: Evidence of local spread and persistence using repeat cross-sectional data

**DOI:** 10.1186/1746-6148-10-95

**Published:** 2014-04-26

**Authors:** Liam J Herbert, Leila Vali, Deborah V Hoyle, Giles Innocent, Iain J McKendrick, Michael C Pearce, Dominic Mellor, Thibaud Porphyre, Mary Locking, Lesley Allison, Mary Hanson, Louise Matthews, George J Gunn, Mark EJ Woolhouse, Margo E Chase-Topping

**Affiliations:** 1 Centre for Immunity, Infection and Evolution, University of Edinburgh, King’s Buildings, Edinburgh, UK; 2 Medical Laboratory Sciences, Kuwait University, Kuwait City, Kuwait; 3 Biomathematics and Statistics Scotland (BIOSS), University of Edinburgh, King’s Buildings, Edinburgh, UK; 4 31A Arthur van Dycklaan, Tervuren 3080, Belgium; 5 Institute of Comparative Medicine, Faculty of Veterinary Medicine, Bearsden, University of Glasgow, Glasgow, UK; 6 Health Protection Scotland, Meridian Court, Cadogan St, Glasgow, UK; 7 Scottish E. coli O157/VTEC Reference Laboratory, Royal Infirmary, Edinburgh, UK; 8 Institute of Biodiversity, Animal Health & Comparative Medicine, College of Medical, Veterinary and Life Sciences, University of Glasgow, Bearsden Road, Glasgow, UK; 9 Epidemiology Research Unit, SRUC, Drummondhill, Stratherrick Road, Inverness, UK

**Keywords:** *E. coli* O157, Epidemiology, Risk factor, Transmission, Persistence, PFGE

## Abstract

**Background:**

*Escherichia coli (E. coli)* O157 is a virulent zoonotic strain of enterohaemorrhagic E. coli. In Scotland (1998-2008) the annual reported rate of human infection is 4.4 per 100,000 population which is consistently higher than other regions of the UK and abroad. Cattle are the primary reservoir. Thus understanding infection dynamics in cattle is paramount to reducing human infections.

A large database was created for farms sampled in two cross-sectional surveys carried out in Scotland (1998 - 2004). A statistical model was generated to identify risk factors for the presence of *E. coli* O157 on farms. Specific hypotheses were tested regarding the presence of *E. coli* O157 on local farms and the farms previous status. Pulsed-field gel electrophoresis (PFGE) profiles were further examined to ascertain whether local spread or persistence of strains could be inferred.

**Results:**

The presence of an *E. coli* O157 positive local farm (average distance: 5.96km) in the Highlands, North East and South West, farm size and the number of cattle moved onto the farm 8 weeks prior to sampling were significant risk factors for the presence of *E. coli* O157 on farms. Previous status of a farm was not a significant predictor of current status (p = 0.398). Farms within the same sampling cluster were significantly more likely to be the same PFGE type (p < 0.001), implicating spread of strains between local farms. Isolates with identical PFGE types were observed to persist across the two surveys, including 3 that were identified on the same farm, suggesting an environmental reservoir. PFGE types that were persistent were more likely to have been observed in human clinical infections in Scotland (p < 0.001) from the same time frame.

**Conclusions:**

The results of this study demonstrate the spread of *E. coli* O157 between local farms and highlight the potential link between persistent cattle strains and human clinical infections in Scotland. This novel insight into the epidemiology of Scottish *E. coli* O157 paves the way for future research into the mechanisms of transmission which should help with the design of control measures to reduce *E. coli* O157 from livestock-related sources.

## Background

*Escherichia coli (E. coli)* O157 is a strain of enterohaemorrhagic *E. coli* (EHEC), also classified as a strain of Shiga-toxin producing *E. coli* (STEC) or verocytotoxin producing *E. coli* (VTEC). Since 1982, *E. coli* O157 has been recognized as an important zoonotic gastrointestinal pathogen of humans. Although the reported incidence is often low, *E. coli* O157 infections are frequently publicized due to large outbreaks and the severity of the illness that it causes, particularly in children and the elderly. Primarily transmitted by the faecal-oral route, infection can arise from animal to human contact, both direct and indirect, human-to-human contact or by foodborne transmission [1].

*Escherichia coli* O157 is the most common reported EHEC serotype in the UK and most countries globally. More than 50 countries have reported cases of human infection with *E. coli* O157, across the 6 inhabited continents [2]. The highest annual incidences of human infection with *E. coli* O157 during the last two decades have been reported in parts of Canada, the United States, Japan and Scotland [3,4]. In Scotland, the mean reported incidence is 4.4 cases per 100,000 population per year (1999-2008) which is consistently higher than which is observed in most other regions of the UK and the world [5].

Cattle are the primary reservoir of *E. coli* O157, and these animals typically have transient asymptomatic infection [6]. Farm-level prevalence of *E. coli* O157 on Scottish beef rearing farms has been estimated at around 21% across farms widely distributed throughout Scotland [7] although a recent study proposed that while ~20% of farms are *E. coli* O157 positive at any one time, in a year >80% of farms will be infected [6]. Widespread agricultural contamination produces a plethora of public health risks. Precautionary principle now requires that all farms in Scotland should be considered to be contaminated [8]. Hence there is a need to minimize the risk of human *E. coli* O157 infection from livestock. This can be achieved through further understanding of transmission.

Natural transmission of *E. coli* O157 between cattle is thought to occur largely through the faecal-oral route, although this may occur indirectly via an environmental reservoir [9]. Persistence and spreading of *E. coli* O157 within farms can be influenced by strain type, duration of shedding, prevalence, magnitude of shedding by individual animals and bacterial survival and growth in the farm environment [10]. Faecal shedding in individual cattle is mainly transient [11] and *E. coli* O157 prevalence is known to be highly skewed [12], most cattle groups test negative for the pathogen, but a small proportion shed high numbers of *E. coli* O157 (i.e. super-shedders). Cattle that excrete high numbers of bacteria can be expected to pose a greater risk of infection to other cattle and humans than those excreting bacteria in low numbers [2].

Persistence of *E. coli* O157 is commonly defined at individual animal level owing to the transient nature of cattle carriage. There is previously published information about persistence in different cattle production systems [13-18]. Individual *E. coli* O157 strains have been isolated for as long as 2 years from dairy herds [14], for as long as 10 months on cattle ranges [16] and over the entire feeding period on cattle feedlots [17]. Liebana et al. [18] examined 11 cattle farms across England and Wales and found that most strains (identified using pulsed field gel electrophoresis (PFGE)) were found only on individual farms but some were found on multiple farms. In another longitudinal study of 9 epidemiologically unrelated farms dispersed across England and Wales Liebana et al. [15] found that some clones can be isolated from the same farm over a period of at least 17 months and from the same animal for a period of at least 7 months. To date there has been no equivalent study in Scotland that has examined local spread and persistence. As such information on farm level persistence of *E. coli* O157 populations in Scotland are less understood.

The objectives of this research were twofold. First to examine the risk factors for the presence of *E. coli* O157 on Scottish farms specifically testing hypotheses regarding local spread and the previous status of a farm. Previous research using data generated from survey 2 in this study failed to provide evidence of local spread [6]. However, the associations within that study were based on the spatial clustering of farms with no direct knowledge of the status of local farms in the area. Second, use PFGE profiles to look for direct evidence of local spread and the presence of persistent strains. To our knowledge this is the first study to examine these issues using data from repeated cross sectional surveys and we aim to expand the available evidence into the factors that influence farm-level carriage and persistence of *E. coli* O157.

## Results

### Agreement of farm status at both time points

The majority of farms (63%) were negative in both surveys (n = 283/447) (Table 1). Thirty-one percent of the farms changed status either positive to negative (17.2%, n = 77/447) or negative to positive (13.4%, n = 60/447). Only 6% of the farms (n = 27/447) were positive for *E. coli* O157 in both surveys. There was no change in the proportion of positive farms between the two surveys (McNemar’s test, p = 0.171).

**Table 1:** **Farm *E. coli *** **O157 status**

		**Survey 2**		
		**Positive**	**Negative**	**Total**
Survey 1	Positive	27	77	104
	Negative	60	283	343
	Total	87	360	447

Status of Scottish farms (positive or negative) during Survey 1 (SEERAD, 1998-2000) and Survey 2 (IPRAVE, 2002-2004). A positive farm was one in which at least one faecal pat tested positive by Immuno-magnetic separation (IMS) for the presence of *E. coli* O157.

### Analysis of risk factors: single variate and multiple variate results

Fourty-nine variables were screened as possible risk factors for this study (Additional file 1: Table S1). Twenty-two variables which had a p value of <0.2 (Table 2) were carried forward to multiple variate analysis. Of the 22 variables, only 2 remained significant as single variables in the multiple variate analysis: the total number of cattle on the farm (p < 0.001) and the number of movements onto the farm in the 8 weeks preceding the sampling in Survey 2 (p = 0.035) were significant risk factors for farm carriage of *E. coli* O157 (Table 3). The previous status of the farm in Survey 1 was not a significant predictor (p = 0.398) of whether or not a farm was positive in Survey 2. Cluster positivity, i.e. another (local) farm in the Survey 2 sampling cluster being positive for *E. coli* O157 was significant both as a single variable (p < 0.001) and in an interaction with Animal Health District (AHD) (p = 0.004). It appears that having a local farm that is positive may only be considered a risk factor in certain AHDs including the Highlands, North East and South West of Scotland. The overall odds ratio for this model was 2.61, with a standard deviation of 0.79. The model shows no evidence of lack of fit (Hosmer-Lemeshow goodness-of-fit chi square = 6.1825 df = 8 p = 0.627). The discriminatory power of the model, represented by the AUC statistic was 0.786. An AUC between 0.7-0.8 gives a fair discriminatory power [19].

**Table 2:** Single variate analysis of risk factors

**Variable**	**Odds Ratio (95%CI)**	**p**
Farm positive Survey 1	1.68 (1.00-2.83)	0.051
Farm in sampling cluster positive	2.97 (1.83-4.81)	<0.001
Season		0.162
Spring	1.52 (0.71-3.25)	0.277
Summer	2.23 (1.08-4.86)	0.298
Autumn	1.89 (0.90-3.95)	0.091
Winter	-	-
Animal Health Division		0.137
Island	0.21 (0.07-0.66)	0.008
Highland	0.75 (0.35-1.61)	0.460
North East	0.63 (0.29-1.35)	0.231
Central	0.61 (0.28-1.34)	0.215
South East	0.81 (0.43-1.84)	0.749
South West	-	-
Management: dairy	2.23 (1.26-3.96)	0.006
Management: beef cattle	0.57 (0.36-0.93)	0.023
Management: change	1.93 (0.98-3.80)	0.058
Pigs present	2.63 (0.92-7.45)	0.070
Farm size: large area (>100km^2^)	1.57 (0.97-2.56)	0.069
Farm size: farm area (km^2^)	1.93 (1.00-3.61)	0.040
Total number of cattle	3.85 (2.04-7.26)	<0.001
Farms within 1 km	1.53 (0.90-2.61)	0.120
Any movement^a^ (open vs closed)	2.54 (0.98-6.60)	0.057
Movement within 1 week of sampling	2.40 (1.24-4.64)	0.010
Movement within 2 week of sampling	2.01 (1.10-3.68)	0.023
Movement within 3 week of sampling	1.88 (1.07-3.32)	0.029
Movement within 4 week of sampling	2.24 (1.33-3.76)	0.003
Movement within 8 week of sampling	2.10 (1.29-3.41)	0.003
No. cattle moved within 4 weeks of sampling	2.94 (1.38-6.23)	0.005
No. cattle moved within 8 weeks of sampling	2.57 (1.41-4.68)	0.002
Arable agricultural^b^	1.66 (0.90-3.08)	0.108
Any *E. coli* non O157 present	1.73 (1.06-2.80)	0.027

Variables identified from single variate logistic regression models for the presence of *E. coli* O157 on the 447 Scottish farms that were sampled in both Survey 1 (SEERAD, 1998-2000) and Survey 2 (IPRAVE, 2002-2004).

^a^ Movement (as defined by Cattle Tracing System (CTS) [20]) is deemed to have occurred when an animal is moved on to a specific farm or between herds.

^b^ Arable agriculture (Land Capability for Agriculture class 1-3.1). Prime agricultural land capable for being used to produce a wide range of crops. Favourable climate; slopes are no greater than 7 degrees; soils are at least 45 cm deep; imperfectly drained [21].

**Table 3:** Multiple variate analysis of risk factors

**Predictor**	**Estimate**	**SE**	**p**
Farm positive in Survey 1	0.264	0.3120	0.398
Season			0.680
Spring	0.132	0.4293	0.758
Summer	0.478	0.4198	0.254
Autumn	0.220	0.4218	0.603
Winter	-	-	-
Farm in sampling cluster positive	1.719	0.6085	<0.001
Animal Health Division (AHD)			0.686
Island	−0.907	0.8698	0.298
Highland	0.128	0.6842	0.852
North East	−0.553	0.7340	0.451
Central	0.555	0.6139	0.367
South East	0.827	0.5956	0.166
South West	-	-	-
Farm in sampling cluster positive*AHD			0.004
Farm in sampling cluster positive in Islands	2.215	1.1426	0.053
Farm in sampling cluster positive in Highland	1.801	0.6558	0.006
Farm in sampling cluster positive in North East	2.441	0.7202	0.001
Farm in sampling cluster positive in Central	−0.373	0.6894	0.589
Farm in sampling cluster positive in South East	−0.417	0.5948	0.484
Farm in sampling cluster positive in South West	1.719	0.6085	0.005
Total number of cattle^a^	1.401	0.3916	<0.001
No. of movements onto farm in last 8 weeks^a^	0.774	0.3658	0.035
**Overall OR**^**b**^	**2.61 (0.79)**		

Results of the logistic regression model of risk factors for the presence of *E. coli* O157 on the 447 Scottish farms that were sampled in both Survey 1 (SEERAD, 1998-2000) and Survey 2 (IPRAVE, 2002-2004). Overall OR gives empirical estimate of odds ratio for the entire model.

^a^log_10_ transformed.

^b^Mean (SD).

### Local spread and persistance

The 87 positive farms from Survey 2 (Table 1) were distributed within 65 of the 161 sampling clusters, and from these farms 500 separate *E. coli* O157 isolates were recovered. There were 21 clusters in which two or more farms were positive; 20 had 2 farms positive, 1 cluster had all 3 farms positive. These data were used to test the hypothesis that *E. coli* O157 could spread between farms in close proximity. Isolates sharing the same phage type (PT) on different farms were found in 10 of the 21 clusters (48%). This was not significantly different from random (10/21 vs 3901/10,000; p = 0.15). PT 21/28 was the phage type identified in 9 of the 10 (90%) clusters where the phage type was the same. PT21/28 was the most common phage type identified in this study (n = 252/500, 50%). As there are so few PTs (n = 12 different types), by random many are the same. Isolates with indistinguishable PFGE profiles were detected in 4 of the 21 (19%) sampling clusters. This is significantly different from random (4/21 vs 151/10,000; p < 0.001).

**Figure 1:**
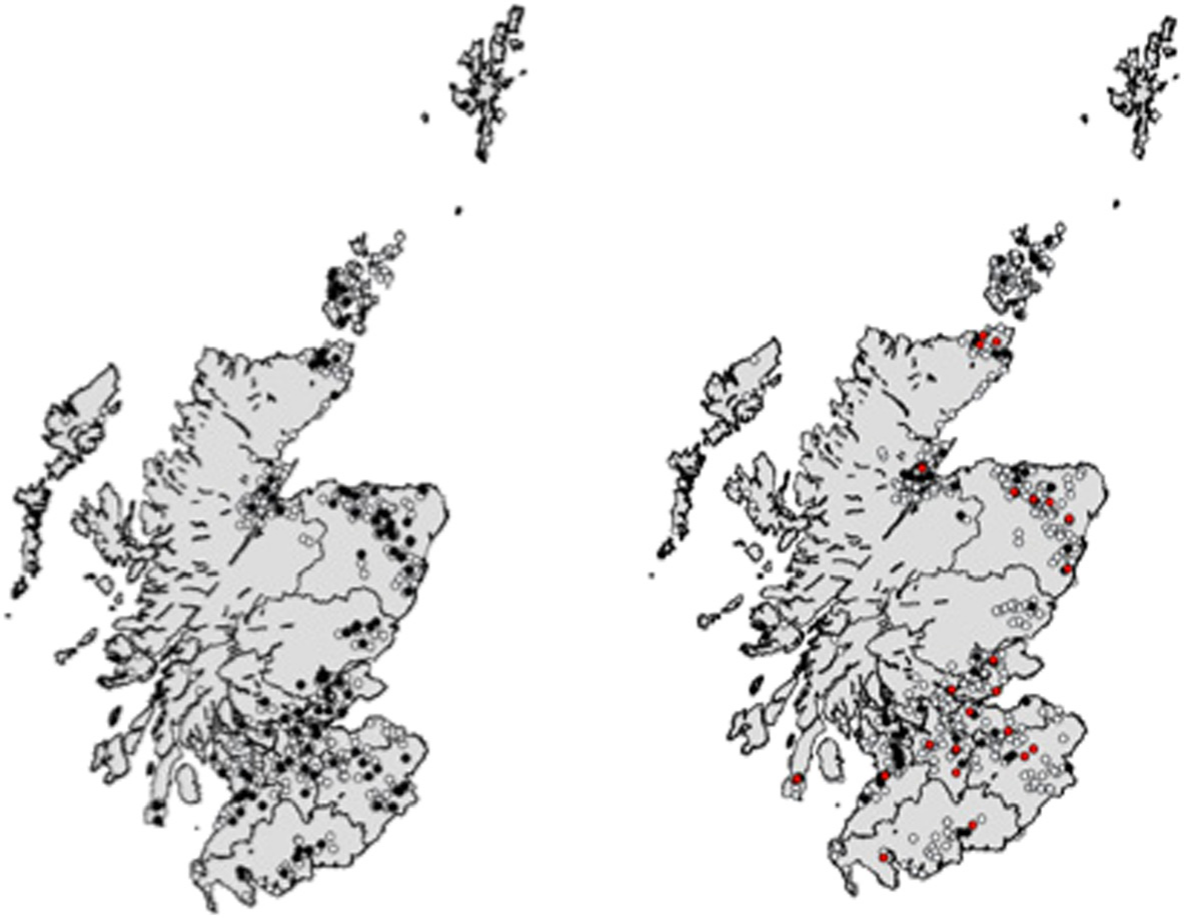
**The geographical distribution of Scottish farms from both Survey 1 (SEERAD, left) and Survey 2 (IPRAVE, right) surveys (n = 447). Left:** Survey 1 (SEERAD, 1998-2000). White circles represent *E. coli* O157 negative farms, black circles represent *E. coli* O157 positive farms. **Right:** Survey 2 (IPRAVE, 2002-2004). White circles represent O157 negative farms, black circles represent *E. coli* O157 positive farms and red circles represent the 27 farms that were *E. coli* O157 positive in both surveys.

Across the 447 farms sampled in both surveys, 139 different PFGE profiles were identified of which 12 (8.6%) were common to both surveys, but not necessarily common to a particular farm. Figure 1 shows the distribution of the 139 PFGE profiles across the two surveys. The first 12 PFGE profiles are those that are present in both surveys (i.e. persistent). Of the 12 persistent PFGE profiles, only 2 (PFGE designated profiles 2 and 3) are well represented in both surveys. Diversity of the PFGE profiles (inset Figure 1) was significantly higher for Survey 2 (the IPRAVE survey) by every measure examined (species richness (SR), Shannon entrophy (SE), Simpson diversity (SD) and Berger Parker (BP)). PFGE profiles marked with an asterisk represent those that were observed in humans (L. Vali unpublished data). Persistent strains were more likely than chance to be those that were identified in humans (Chi-square test, p < 0.001). Six of the 12 strains (50%) identified as present in both surveys were also observed in human clinical infections.

Although persistent strains were identified they were not necessarily identified on the same farm. Of the 27 farms that were positive for *E. coli* O157 in both Survey 1 and Survey 2 (Table 1), 24 had PFGE profiles for isolates from each survey. The number of farms with the same PFGE profile present on a farm at the two time points (2 farms out of 24) was compared to a large bootstrap sample of all farms in the two surveys where two different farms were randomly paired (444 out of 10,000). These proportions were not significantly different (p = 0.090).

## Discussion

To our knowledge, the surveys examined in this study represent the only reported systematic national surveys of bovine *E. coli* O157 shedding and present a valuable opportunity to simultaneously examine factors influencing cattle carriage and persistence of *E. coli* O157 at farm level. Although the repeated cross-sectional design has been employed for analysis of *E. coli* O157 previously [22], we know of no other paper on the topic with the additional benefit of molecular analysis. Furthermore, the use of PFGE data in this study is also advantageous as PFGE methods have been used previously to show persistent and local spread in other *E. coli* O157 research [17,23,24].

This study analyses a very large database with risk factors representing farm size, composition and clustering, farm management, feed, infection status, and landscape type (Additional file 1: Table S1). Despite the comprehensive nature of the available information, no risk factors associated with feed, management or the presence of other livestock were identified in the final model. In addition there is no evidence that the previous status of the farm is important. This agrees with the conclusion of Zhang et al [6] which suggested that in a given year approximately 80% of the farms in Scotland will likely have cattle that are shedding *E. coli* O157. It also reaffirms the position of public health officials who have recommended that all farms in Scotland should be considered positive [8]. In keeping with a prior analysis of the Survey 2 (IPRAVE) data the risk factors identified included the size of the farm (total number of cattle) and recent movements (represented here by the number of cattle moved onto the farm within 8 weeks of sampling). The absolute odds ratio (SD) from the Zhang et al. [6] paper was only 1.297 (0.379). The addition of risk factors pertaining to local spread (local farm positive) increased the odds ratio (SD) of the overall risk model to 2.61 (0.79). Zhang et al. [6] found no evidence of local spread although this was not tested directly as we have done in this study. It may be the case, however, that local spread is only of increased risk within certain regions as suggested by the interaction of AHD and local farm positive. Farms in areas of North East, Highland and South West were significantly more at risk if a local farm was identified as positive. This may reflect differences in biosecurity within these regions. Although the importance of spatially variable factors such a distance between farms (neighbours can be further apart in some area than others) [25] and topography cannot be discounted.

Two important findings within this study are evidence of local spread and persistence. Indistinguishable PFGE profiles were present on local farms, suggesting local farm-to-farm spread of *E. coli* O157 strains, and on farms after a period of approximately 3 to 4 years, suggesting persistence. These results are of even greater importance as with only one colony per pat being analysed our results are likely to underestimate the number of common strains. In addition, we only considered strains with indistinguishable PFGE profiles, not variants differing by one or two bands by PFGE. Evidence of local spread of *E. coli* O157 has been reported previously, [26]. However, literature suggesting frequent transmission between farms is rare. This study adds weight to a growing body of evidence, concurring with the results of two North American studies by Wetzel *et al*[27] and Rosales-Castillo *et al*[28]. The former found genetically indistinguishable isolates on neighbouring Ohio dairy farms whilst the latter reported the spread of *E. coli* O157 isolates between Mexican cattle farms. The results of our molecular analysis however cannot determine whether the spread of strains between farms is direct, due to factors such as cattle or human movement, or whether the indistinguishable strains we found come from a pool of environmentally persisting strains that are spread indirectly between farms through the movement of contaminated feed or wildlife, for example wild birds [27].

The presence of *E. coli* O157 on the closest local farms (farm in sample cluster positive) is a plausible risk factor as farms traditionally do not operate in isolation and farm staff within a locality may well visit other farms with some regularity, as well as using shared resources such as feed delivery or milking trucks. *E. coli* O157 could be transmitted to a new farm either through exchange of animals or by being transmitted by humans via foot or vehicle. The farm management questionnaire used in both Survey 1 and 2 did not ask about contacts with other farms so this potential transmission network cannot be tested formally. However, Rosales-Castillo *et al*[28] identified milking and other staff movement as a risk factor for farm-to-farm transmission. An alternative hypothesis could be that the clustering implies proximity to a shared environmental reservoir. In either case, cluster positivity encompasses the spread of *E. coli* O157 strains to and from local farms or an environmental reservoir.

**Figure 2:**
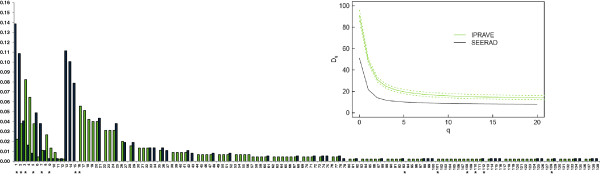
**Distribution of the PFGE types observed in Survey 1 (SEERAD, black bars) and Survey 2 (IPRAVE, green bars)**. Distribution of PFGE types is organized so that the first 12 represent PFGE types that are common to both the field surveys. Asterisks represent PFGE types known to be present in human clinical samples for the same time frame. Inset graph is the diversity profiles for the Survey 2 (IPRAVE, green) and Survey 1 (SEERAD, black) surveys illustrating the significantly higher diversity of PFGE types observed in Survey 2 (IPRAVE). Dotted lines represent the confidence intervals for Survey 2 (IPRAVE) data generated by subsampling to the size of Survey 1 (SEERAD).

Persistence of *E. coli* O157 PFGE profiles has been reported in the literature but the majority of this research was conducted as longitudinal studies [13,15,17,18,23] or repeated cross sectional surveys [10] of the same cohorts. Within this research *E. coli* O157 strains were observed to persist across the two surveys (n = 12, Figure 2) as well as on the same farm (n = 3) after 3-4 years and turnover of cattle. This result suggests that there may be certain strains within Scotland that have found a niche within the cattle environment. This is of concern as there seems to be an association with the existence of persistent cattle strains and the strains that are observed in human *E. coli* O157 cases.

## Conclusions

Our findings highlight the relevance of local spread and persistent strains of *E. coli* O157 on Scottish cattle farms. The significance of local spread within certain AHDs is suggestive of biosecurity and or spatial geographical differences within Scotland. Further research is being conducted to determine biosecurity throughout Scotland and the importance of local geography on pathogen spread. Results of these studies may shed some light on the results of this research. The observation that there may be persistent strains within Scotland that are contributing to the number of human cases reported warrants further investigation. This is currently being investigated in a Scottish research collaboration.

## Methods

### Data sources

Data from multiple sources were pooled for use in this study including the following: (1) the June 2003 Agricultural census of livestock premises combined with the department for Environment, Food and Rural Affairs (DEFRA) list of livestock premises; (2) the Cattle Tracing System (CTS), (3) Land Capability for Agriculture in Scotland; (4) *E. coli* non-O157 prevalence data; (5) *E. coli* O157 prevalence data, (6) *E. coli* O157 typing and PFGE data. Details on most of the data sources have been published previously however, each data set will be described briefly below.

(1) June 2003 Agricultural census data [29]. Among the original 50,266 farms in Scotland recorded in the census, 22,286 farms are provided with the numbers of animals but only 13,704 farms have cattle. Our system comprises these 13,704 cattle farms. The data include the Council-Parish-Holding number (CPH), the X-Y coordinates of the farm-house, the area of the farm, and the numbers of cattle, sheep and pigs. The number of cattle on each farm is assumed constant at the number recorded in the census.

(2) Cattle Tracing System (CTS). The CTS is operated by DEFRA’s British Cattle Movement Service [20,30]. In Scotland, during years 2002-2004 there were 252,496 movements among 11,464 of the cattle farms entered in the 2003 census database (the remainder are assumed not to have moved cattle to or received cattle from other farms). Movements outside Scotland and to/from abattoirs and markets are not considered here.

(3) Land Capability for Agriculture in Scotland (LCA). The LCA classification was developed by the Macaulay Institute to describe the agricultural potential of land based on the degree of limitation imposed by its biophysical properties. It is based primarily on climate, a number of soil properties, (for example depth and stoniness), wetness, erosion risk and slope. Also included are the overall pattern, i.e. variability, and, in one of the classes (Class 6), vegetation cover is also taken into account. The LCA is a seven class system where class 1 represents land that has the highest potential flexibility of use whereas class 7 land is of very limited agricultural use [21].

(4) *E. coli* O157 prevalence data. Between March 1998 and February 2004 two cross sectional surveys were conducted in Scotland. Data used in this study came from 447 farms sampled in both surveys (Figure 2). The first survey (Survey 1) was conducted by the Scottish Agricultural College through funding from the Scottish Executive Environment and Rural Affairs Department (SEERAD) from March 1998 to May 2000. The second survey (Survey 2) was carried out between February 2002 and February 2004, funded by the Wellcome Trust International Partnership Research Award in Veterinary Epidemiology (IPRAVE). The field sampling methodologies for both surveys have been described in previous literature [7,12], however, a brief outline is given below. Further, farmers were asked to complete a farm management questionnaire from which much of the data about the farms were gathered. Prior to sampling, written consent was obtained from the farmers for participation in the study.

Both surveys preferentially sampled cattle groups composed only of store (i.e. weaned cattle before finishing for slaughter) or finishing cattle closest to sale or slaughter. If such groups did not exist, one or more mixed groups with store or finishing cattle closest to sale or slaughter were sampled. From each group fresh faecal pats were sampled. The number of pats tested in each group was determined from the number of cattle in the group using a prescribed sampling schedule. For Survey 1, sufficient numbers of faecal pats were tested to ensure prospectively an 80% chance of sampling at least one positive pat if there was a shedding prevalence of at least 2% within the group [12]. Based on results from Survey 1, in Survey 2, it was assumed that, on average, 8% of the animals in positive groups would be shedding, with shedding distributed as seen in Survey 1 [7]. For each group in Survey 2, sufficient fresh pat samples were taken to ensure prospectively a mean 90% probability of detecting shedding of *E. coli* O157 if at least one shedding animal was indeed present. Changes in sampling strategy between the two surveys had a negligible effect on the power to identify positive farms [7]. Instead of randomly sampling farms within each Animal Health District (AHD), Survey 2 used a stratified sampling plan derived from the Survey 1 cohort to select farms to sample [31]. Farms were selected randomly then the farms with closest Euclidean distances were sampled on the same or concurrent days, leading to clusters of 3 independent farms. The sampling of the same farms between the studies and the comparable methodology between the studies allow the use of these separate surveys as two cross-sectional time points for our analysis.

(5) *E. coli* non O157 prevalence data [32]. Collection of bovine isolates for the detection of *E. coli* O26, O103, O111 and O145 done in parallel with Survey 2 (2002-2004). Fecal samples were taken from 338 farms to test for non-O157 *E. coli* strains as described in [32].

(6) *E. coli* O157 typing data. Within 48 hours of sampling, one gram of faeces from each pat sample was tested for the presence of *E. coli* O157 through immuno-magnetic separation (IMS) and culturing as described in detail elsewhere [33]. Following IMS, one *E. coli* O157 isolate from each faecal sample was submitted to the Scottish *E. coli* O157/VTEC Reference Laboratory (SERL) for phage typing, and testing for the presence of genes encoding the virulence factors shigatoxin 1 (*stx*_1_), shigatoxin 2 (*stx*_2_) and intimin (*eae*) using multiplex PCR. PFGE analysis was conducted on *E. coli* O157 isolates from both surveys as described previously [33] as well as randomly selected isolates selected from human clinical samples from the same time frame. Briefly, isolates were digested with 50U of *XbaI* restriction enzyme, then subjected to PFGE using CHEF DRII apparatus (Bio-Rad laboratories, UK). Further analysis and categorization of PFGE results were conducted using Bionumerics 4.1 (Applied Maths, Belgium).

### Statistical analysis

#### Agreement between surveys

The presence and absence of agreement between farm *E. coli* O157 status on farms in Survey 1 and Survey 2 was compared using McNemar test. McNemar’s test assesses the significance of the difference between two correlated proportions. The analysis was performed using StatXact version 8 (Cytel Software Corp, Cambridge, MA, USA). The null hypothesis is that the proportion of farms with the characteristic (or event) is the same for Survey 1 and Survey 2.

### Risk factor analysis

Data from all of the sources listed above was compiled and used as the basis of the risk factor analysis. Risk factors for the presence of *E. coli* O157 on a farm were analysed using logistic regression analysis (Proc Logistic, SAS Institute Inc., Cary, NC). Logistic regression analyses were carried out on a single variate basis initially. All the potential risk factors (Additional file 1: Table S1, n = 49) were examined. All variables with a p value of <0.2 were retained for multiple variate analysis. Region and season were forced into the model as design factors. Seasons were defined as winter, comprising December, January, and February; spring, comprising March, April and May; Summer, comprising June, July and August; and Autumn, comprising September, October and September. Six regions, based on Veterinary Animal Health Districts (AHDs) were defined: 1 = Islands; 2 = Highland; 3 = North East; 4 = Central; 5 = South East; 6 = South West. A hierarchical forward selection and backward elimination approach with swapping (reassessment of previously included or excluded variables) were used. The change in the deviance of the model was monitored as an indicator of improved fit. Variables were added and removed based on significant improvement in the mean deviance after changes to the model. Two-way interactions were also tested in this manner. The inclusion of a random effects term for cluster did not improve the fit of the model significantly.

Model fit was assessed by fitting ROC curves to the final models and generating area under the curve (AUC) statistics for the models. The AUC can be considered to be a measure of the discriminatory power of the model [34]. A theoretically perfect model would have an AUC = 1 while a model with no discriminatory power would have AUC = 0.5. Thus using this scale, the AUC statistics of the models and hence the probability of the model being able to discern between a positive and negative *E. coli* O157 farm can be assessed and compared. For the final model, the Hosmer-Lemeshow goodness-of-fit statistic was computed [19].

To check for multicollinearity between factors in the final model, correlations were examined for binary and nominal variables. In addition, the stability of the model was checked by systematic removal of variables. Diagnostics were performed and plots of residuals were examined, confirming goodness of fit of the model. Odds ratios and their associated 95% CI were estimated in the final model for factors statistically significantly associated with the presence of *E. coli* O157.

To compare the results of the statistical analysis in this study to those generated earlier by Zhang et al. [6] an overall odds ratio estimate that was developed for the earlier study was calculated. Values for the empirical estimate of the odds ratio were derived using the parameter estimates from the logistic regression model. These estimates were used in the statistical model to simulate binary response random variables for each farm (absence/presence of *E. coli* O157 on-farm). The predicted presences/absences were then related to the observed presences/absences, and aggregated over all farms, from which summaries an odds ratio was calculated by

(1) $$\mathrm{OR}=\frac{F_{++}\times F_{--}}{F_{+-}\times F_{-+}}$$

where *F*_++_ is the number of farms that are positive for both model prediction and observed data, and the meanings of *F*_−−_, *F*_+-_ and *F*_-+_ follow accordingly [6]. An odds ratio greater than 1 indicates that the model is more likely than not to predict the correct infection status of a farm. The larger the odds ratio, the stronger the predictive power of the model. This process was repeated to produce a distribution of odds-ratios which could then be summarised.

### Local spread and persistence

Survey 2 sampling clusters in which >1 farms were positive were investigated to determine whether any identical *E. coli* O157 strains could be found on farms within the vicinity of each other (average distance = 5.96km). BioNumerics 4.1 software (Applied Maths, Belgium) was used to analyse the PFGE profiles. Dendrograms were generated using unweighted pair group method with arithmetic mean (UPGMA) with settings of 1.00% optimisation and 1.3% position tolerance and provided a visual representation of the relationships among isolates. Each unique PFGE profile was allocated a profile identifying code (1-139). For the purposes of our local spread analysis, isolates had to be indistinguishable (ie 100% similar) for classification as locally spreading. Analysis determined whether sampling clusters were more likely to have the same PT / PFGE profile than random. To do this the clustered data were compared to 10,000 bootstrap samples of pairs of positive farms sampled at random.

Farms that were *E. coli* O157 positive in both Survey 1 and Survey 2 (n = 27, Table 1) were analysed using the same dendrogram protocol. This analysis was conducted to identify any strains that were present in both Survey 1 and Survey 2. The criteria for being defined as the same strain was 100% similarity using the above dendrogram protocol. This is a conservative estimate and likely a lower estimate as it does not allow for common strain development.

### Ecological diversity of PFGE types

Diversity of PFGE types in both surveys was examined using multiple diversity measures, related to Renyi’s measures of generalized entrophy [35,36] similar to the analysis done in Mather et al. [37]. The exponential of Renyi’s entrophy measure gives an estimate of the effective number of species *D*_*q*_[38,39], with its single parameter *q* determining the extent to which rare PFGE types (in this instance) contribute towards overall diversity (Equation 2). The following diversity indicies were calculated for both surveys: specie richness (SR), D_0_; Shannon Entrophy (SE), log(D_1_); Simpson diversity (SD), 1/D_2_; and Berger-Parker (BP), 1/D_∞_

(2) $$D_{q}\left(p_{1}Kp_{s}\right)=\left\{\begin{array}{ll} {\left[\Sigma_{i=1}^{s}p_{i}^{q}\right]}^{1/1-q}, & q\neq 1,\\ \prod_{i=1}^{s}pi^{‒pi}, & q=1 \end{array}\right)$$

While it is trivial to say that values of D_0_ would inform on the total number of different PFGE types present in the survey data, values of D_∞_ would carry out some information on the number of PFGE types that seems to dominate the profiles observed.

To measure the diversity of each sample (Survey 1 and Survey 2), true abundances of the sample were derived directly from the count data (p_j_ = n_j_/Σn_j_), and the different diversities calculated from the abundance proportions. However, because sample diversity measures depend heavily on sample size [40], direct comparison was conducted by repeatedly subsampling the larger (Survey 2) sample to the size of the smaller (Survey 1) sample with replacement. The algorithm was applied 1000 times to generate 1000 different sub-dataset of similar size as Survey 1 and enabling the creation of confidence intervals around D_q_ and, therefore, every every measure examined. Analysis was conducted in R version 3.0.2 (R Development Core Team, 2013) [41].

## Competing interests

We declare that none of the authors at the time of the study or preparation of the paper have any competing interests that could influence or bias the content of this paper.

## Author’s contributions

LJH conducted the initial statistical analysis and wrote the initial manuscript. LV and DVH performed the PFGE analysis. GJG co-ordinated the field sampling. MCP and DM conducted field sampling. GI helped with the statistical analysis of PFGE profiles. IJM provided the code to calculate the absolute odds ratio. TP generated the diversity profiles. LA performed the phage typing, LA and MH provided the clinical isolates for the study and ML provided information on human case data in Scotland. LM contributed to final manuscript. MEJW co-ordinated the epidemiological study and contributed to final manuscript. MECT created the dataset and performed the final statistical models and made the revisions required for submission of the final manuscript. All authors read and approved the final manuscript.

## Supplementary Material

Additional file 1: Table S1 List of all risk factors examined in this study. The 49 variables are grouped according to the broad categories. C, this variable was treated as a categorical variable for statistical analyses; Q, this variable was treated as a quantitative variable for statistical analyses. Click here for file
