# Supplementary material for: E. coli O157 on Scottish cattle farms: Evidence of local spread and persistence using repeat cross-sectional data
Source: BMC Vet Res. 2014 Apr 26;10:95. doi: 10.1186/1746-6148-10-95 (PMC4022360; doi:10.1186/1746-6148-10-95)
Supplement: Additional file 1: Table S1 — List of all risk factors examined in this study. The 49 variables are grouped according to the broad categories. C, this variable was treated as a categorical variable for statistical analyses; Q, this variable was treated as a quantitative variable for statistical analyses. [file 1746-6148-10-95-S1.docx]

**Additional file**

Table S1. List of all risk factors examined in this study. The 49 variables are grouped according to the broad categories. C, this variable was treated as a categorical variable for statistical analyses; Q, this variable was treated as a quantitative variable for statistical analyses.

| ***Hypothesis variables:***  Farm Status SEERAD (C)  Farm in cluster positive (C) |  |
| --- | --- |
| ***Study Design variables:***  Season (C)  Animal Health Division (AHD) (C) |  |
| ***Farm size:***  Total number of cattle (C,Q)  Number of finishing cattle (C,Q)  Farm area (C,Q) | ***Cattle movement:***  Any movement (open v closed) (C)  Number of movement events (C,Q)  Movement in 1 week (C,Q)  Movement in 2 week (C,Q)  Movement in 3 week (C,Q)  Movement in 4 week (C,Q)  Movement in 8 week (C,Q) |
| ***Farm management:***  Dairy (C)  Cattle (C)  Change in management (C)  Location: Housed (C)  Location: Grazing (C) |  |
| **Farm composition:**  Age of cattle (C,Q)  Number of sheep present (C,Q)  Pigs present (C)  Chickens present (C)  Dogs present (C)  Cats present (C)  Horses present (C)  Wild geese present (C)  Wild gulls present (C) | ***Farm infection status:***  *E. coli* O26 present (C)  *E. coli* O145 present (C)  *E. coli* O103 present (C)  Any non *E. coli* O157 infection (C) |
| ***Farm clustering:***  Farms within 1 km (C)  Number of farms within 3km (Q)  Number of farms within 5 km (Q) |  |
| ***Water:***  Mains (C)  Private (C)  Natural (C)  Change to mains water supply from private (C)  Change to private water supply from mains (C) | ***Feed (other):***  Turnips (C)  Brewers grains (C)  Dark grains (C)  Draff (C)  Minerals (C) |
| **Land Capability (spatial descriptors):**  LCA code (C)  Arable land (C)  Mixed land (C)  Rough land (C) |  |
